# On-body electrochemical measurement of sweat lactate with the use of paper-based fluidics and 3D-printed flexible wearable biosensor

**DOI:** 10.1007/s00216-025-05905-0

**Published:** 2025-05-09

**Authors:** Gabriella Iula, Antonella Miglione, Panagiota M. Kalligosfyri, Michele Spinelli, Angela Amoresano, Concetta Di Natale, Ibrahim A. Darwish, Stefano Cinti

**Affiliations:** 1 Department of Pharmacy, University of Naples ‘Federico II’, Via D. Montesano 49, 80131 Naples, Italy; 2 Department of Chemical Sciences, University of Naples “Federico II”, 80126 Naples, Italy; 3 Department of Chemical Materials and Industrial Production (DICMaPI), University of Naples Federico II, P.le Tecchio 80, 80125 Naples, Italy; 4 Department of Pharmaceutical Chemistry, College of Pharmacy, King Saud University, P.O. Box 2457, 11451 Riyadh, Saudi Arabia; 5 Bioelectronics Task Force at University of Naples Federico II, Via Cinthia 21, 80126 Naples, Italy; 6 Sbarro Institute for Cancer Research and Molecular Medicine, Center for Biotechnology, College of Science and Technology, Temple University, Philadelphia, PA USA

**Keywords:** 3D-printing, Paper-based, Screen-printed electrodes, Lactate, Wearable biosensors

## Abstract

Real-time monitoring of sweat lactate provides valuable physiological insights for assessing exercise outcomes and athletic performance. Conventional lactate detection methods, while sensitive, often lack portability and real-time capability for use in wearable or in-body applications. To address these limitations, electrochemical biosensing has emerged as a leading approach, enabling non-invasive and real-time analysis. Wearable devices which integrate lactate-specific enzymes with electrochemical transducers might provide efficient solutions for continuous monitoring. In this study, a wearable lactate biosensor was developed using custom screen-printed electrodes modified with a bio-hybrid probe comprising Prussian blue, carbon black, and lactate oxidase. All the key experimental parameters were optimized, and a detection limit of 60 µM and a linearity up to 20 mM were obtained. A filter paper-based strip was incorporated to enhance sweat collection and serve as the real sample collector by exploiting its porosity: this configuration allowed a satisfactory repeatability of 6%. The system was validated using real sweat samples, highlighting a quantitative correlation (94–103%) with LC–MS/MS measurements. The biosensor was integrated onto a 3D-printed thermoplastic polyurethane (TPU) armband, designed for a customizable and comfortable fit, ensuring effective sweat collection and transport. This low-cost, wearable system represents a significant step forward in non-invasive, continuous, and personalized health monitoring, providing a practical tool for tracking physiological parameters in real-time.

**Supplementary Information:**

The online version contains supplementary material available at 10.1007/s00216-025-05905-0.

## Introduction

Lactate, a by-product of anaerobic metabolism, plays a crucial role in sport and physical activity. During high-intensity exercise, oxygen demand exceeds oxygen supply, increasing glycolysis and producing lactate from pyruvate. Previously viewed only as a fatigue-inducing waste product, lactate is now recognized as an essential energy substrate and signaling molecule. Lactate is known to shuttle between cells (the “lactate shuttle”) and organs, such as from muscles to the liver via the Cori cycle, where it is converted back to glucose [1, 2]. Normal resting blood lactate levels range from 0.5 to 1.5 mM, but during intense exercise, they can rise to 4–20 mM [3], depending on fitness and exercise intensity. This elevation indicates increased anaerobic metabolism and recruitment of fast-twitch muscle fibers [4]. Lactate is also found in sweat, with concentrations reflecting local and systemic metabolic activity during exercise, typically ranging from 5 to 25 mM based on intensity and duration [3, 4]. While sweat lactate does not directly indicate systemic anaerobic metabolism, elevated levels may reflect local muscle effort and sweat gland activity, serving as a useful non-invasive marker for metabolic status. The levels of sweat lactate are of practical significance in sports science, allowing for non-invasive monitoring of metabolic responses and potentially aiding in the optimization of training and recovery strategies [5].

Traditional lactate sweat monitoring methods rely on laboratory techniques for collecting and analyzing sweat samples. Common approaches include mass spectrometry, which quantifies lactate but requires extensive processing and is unsuitable for real-time monitoring. Other methods, like high-performance liquid chromatography and colorimetric techniques, typically require external sweat collection prior to analysis, which can affect the integrity of the sample [6]. Sweat is often collected using methods like whole-body washdown, absorbent patches, or plastic bags, which can influence the results and lack immediacy in measurement [7]. The sweat rate and composition measured through localized methods often vary by body site, failing to accurately reflect overall sweat content and average sweat rate [7]. Traditional techniques for sweat collection are complex and not well-suited for continuous monitoring during physical activities.

While they provide valuable insights into sweat physiology and lactate levels, advancements in improved performance technologies are being explored for more efficient and less intrusive continuous monitoring and collection of sweat data [8]. Among these, electrochemical sensing stands out as a widely used and well-established method for sweat lactate analysis, particularly when the screen-printing technology is combined into wearable sensors [9, 10]. Most lactate biosensor developments have primarily focused on enzymatic sensing methods, utilizing lactate oxidase (LOx) or lactate dehydrogenase (LDH), due to their high specificity and sensitivity [11]. Nonenzymatic methods using materials like metal oxides and molecularly imprinted polymers offer advantages such as lower cost and simpler fabrication. However, they often require highly basic solutions, which do not accurately replicate human biofluids [12–14]. In contrast, enzymatic sensing techniques have been explored for non-invasive monitoring of lactate in sweat, demonstrating significant potential for clinical diagnostics [10, 15, 16]. Their high performance, portability, simplicity, and cost-effectiveness make them particularly attractive for widespread application in health monitoring. Since wearable sweat sensors for real-time lactate monitoring were first introduced in 2013 [17], numerous studies have explored diverse strategies to enhance the accuracy, reliability, and convenience of sweat lactate analysis. For instance, electrochemical tattoo biosensors enable real-time monitoring but may have limited durability [17]. The integration of microfluidic technology addresses many challenges affecting data integrity, enhances sweat collection, and significantly improves sample transport, resulting in better temporal resolution and more accurate sweat analyte measurements [18, 19]. Combining wearable multisensing patches and microfluidic epidermal systems can improve sweat collection and sampling efficiency but can be challenging to integrate with everyday clothing [10]. Eyeglass-based platforms provide discreet usability but face challenges due to limited sweat access. On the other hand, integrated sensing papers and zero-power osmotic sweat extraction systems improve energy efficiency and sample collection but raise concerns about long-term stability [20]. Despite these advancements, balancing sensitivity, user comfort, and reliable data acquisition continues to be a significant challenge. A detailed table (Table S2) comparing several examples of wearable screen-printed electrochemical biosensors is provided in the Supplementary Information file (SI) [17, 20–22].

Building on these considerations, we integrated a simple paper-based microfluidic platform with a flexible screen-printed electrode (SPE) sensor, incorporated into a 3D-printed wearable armband for continuous sweat sampling and monitoring. In particular, in the present work, we would like to emphasize how the development of a flexible home-made SPE modified with a bio-hybrid probe containing Prussian blue (PB), carbon black (CB), and lactate oxidase enzyme (LOx) can be combined with a filter paper strip that enhances sweat collection efficiency while maintaining a lightweight design, ensuring comfort during prolonged wear [23]. All the architecture was successfully housed in a wearable and low-cost 3D-printed armband, printed in thermoplastic polyurethane (TPU), which provides flexibility, durability, and customization, which enhances wearability and comfort. The use of 3D printed TPU ensures a lightweight, flexible, and ergonomically contoured armband. Unlike rigid designs, this improves user comfort during prolonged wear, making it suitable for dynamic activities such as sports or rehabilitation. The bracelet can be easily customized to fit different arm sizes and adapt to the needs of specific populations such as athletes, children or clinical patients. This level of personalization is often lacking in off-the-shelf wearable monitors. TPU’s ability to withstand stretching, bending and environmental stress (e.g., sweat and moisture) ensures long-term reliability, a critical feature for wearable devices that are subjected to rigorous use [24, 25]. By incorporating a tight fit for the potentiostat and SPE, the TPU armband eliminates the need for external attachments, streamlining the design for a compact and seamless user experience; moreover, TPU-based 3D printing reduces material waste compared to traditional manufacturing techniques, supporting sustainability while enabling rapid prototyping and scalability [25].

## Materials and methods

### Reagents and equipment

Lactate Oxidase from *Aerococcus viridans* (100UN) (LOx), sodium L-lactate (LA), iron(III) chloride (FeCl_3_), potassium hexacyanoferrate(III) (K_3_[Fe(CN)_6_]), potassium chloride (KCl), N,N-dimethylformamide (DMF), and phosphate buffer (pH = 7.2; 26.22 g/L monopotassium phosphate, 7.78 g/L sodium carbonate) were purchased from Merck Life Science (St. Louis, MO, USA). Uric acid (UA), dopamine hydrochloride (DA), and glucose (GLC) were purchased from Sigma-Aldrich (St. Louis, MO, USA). All solutions were prepared in Milli-Q water produced in-house to 18 MΩ/cm quality, using a Milli-Q® EQ 7015 Ultrapure Water Purification System (Darmstadt, Germany). Conductive inks (Ag/AgCl and graphite) were purchased from Sun Chemicals (USA). Carbon black (CB) VXC72R powder was purchased by Cabot Corporation (Italy). Flexible polyester film (HT5 Autostat) was kindly provided by McDermid Alpha (UK) and MacDermid Performance Solutions Italiana (Italy). Whatman No.1 chromatography paper was purchased from Cytiva (USA). Adobe Illustrator was used to design the wax pattern of the channel strip on the Whatman No. 1 chromatography paper, printed through a solid ink printer, namely, ColorQube 8580 from Xerox (USA). Sweat samples were voluntarily provided by members of the research team under non-invasive conditions and without the collection of any personal or medical data. All the electrochemical measurements were carried out with the use of a portable potentiostat, Sensit Smart (PalmSens, The Netherlands), connected to a laptop and to an Android smartphone. Current responses were recorded and displayed by using the dedicated application PStouch by Palmsens BV.

### Screen-printed biosensor combined with the filter paper strip

**Fig. 1:**
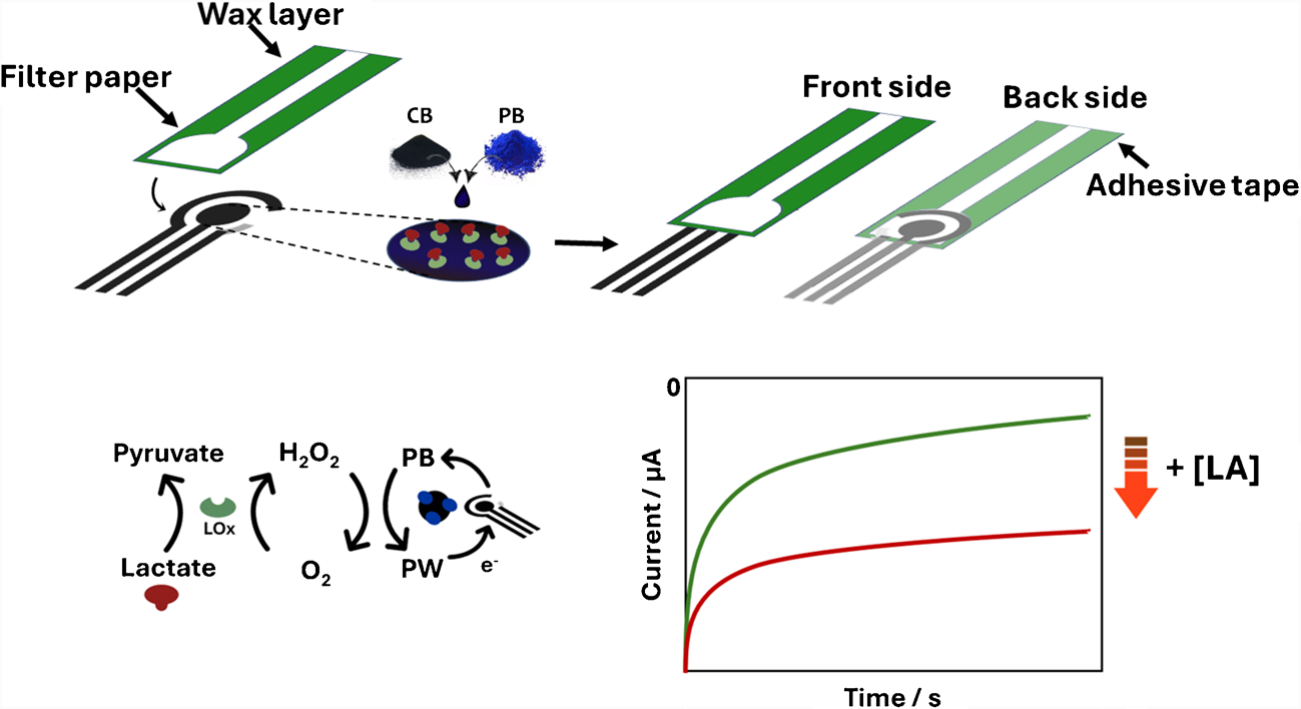
Schematic representation of the enzyme-based SPE integrated with the filter paper strip for sweat lactate monitoring

Electrodes were in-house produced onto a flexible polyester film as substrate. The use of polyester-based substrates for the manufacture of electrochemical strips allows the creation of more robust and flexible platforms that can be used in a decentralized and wearable context. The three-electrode design was screen-printed using a semi-automatic screen-printer equipped with a squeegee to spread the conductive inks through an ad-hoc designed mask. Briefly, Ag/AgCl ink was used to print the connections and the reference electrode, and the carbon ink was used to print the working and counter electrodes. After the inks were printed, the strips were thermally cured, 100 °C for 30 min, necessary to make the printed ink stable for electrochemical measurements. The diameter of the working electrode was 0.4 cm, and the electrochemical strips were ca. 2.5 cm (height) × 1 cm (width). The possible diffusion of aqueous samples to the connector was avoided by placing an adhesive tape to define the testing area [26, 27]. The final architecture in which the SPE, printed on flexible polyester, was combined with the filter paper strip, which was obtained by firstly drawing the strip on Adobe Illustrator and wax-printed onto a Whatman No. 1 chromatography paper. Then, a waxed paper was cured in an oven at 100 °C for 1 min, allowing the wax to diffuse through the paper, producing a hydrophobic layer all around the hydrophilic inlet area. This step is very important to define the sample chamber and confine the solution in the delimited electrochemical cell area [28–30]. The microfluidic channel design was selected based on well-established geometries known to support effective sweat transport via capillary action and passive flow. Our primary aim was to create a design that balances functional efficiency with practical integration into wearable systems. For this reason, we chose a straightforward and compact channel shape that can be easily fabricated and comfortably integrated into a wearable device. The current design reflects a trade-off between simplicity, manufacturability, and reliable performance [28]. Subsequently, the backside of the printing surface was covered with adhesive tape (without covering electrical connections) to prevent the solution leaking out. The complete platform consisted of a hydrophilic rectangular zone (microfluidic channel, 2.6 cm (length) × 0.2 cm (width)), connected to a flexible polyester electrochemical cell, where the electrodes were screen-printed as reported in Fig. 1.

### Biosensor nanoengineering

The electrochemical printed strips have been successively modified with 2µL of the mixture composed of carbon black (CB) and Prussian blue (PB), obtained by directly synthesizing PB on CB, according to the procedure reported by Cinti et al. [31, 32]. The suspension was prepared dissolving 1 mg of the powder in 1 mL of DMF/H_2_O (1:1), sonicated for 1 h, and then drop casted onto the working electrode area and let dry; then 2 µL of the 25 U/mL enzyme, LOx, has been drop-cast and let dry (Fig. 1) [33, 34].

### Electrode characterization

Electrodes were morphologically characterized using a Hitachi TM3000 Scanning Electron Microscope (SEM). Energy-Dispersive X-ray Spectroscopy (EDX) was performed using the SwiftED 3000 X-Stream module (Hitachi) in combination with SEM. This analysis enabled the detection of Fe in the Prussian blue-coated electrodes and allowed comparison with the bare electrode surfaces.

### Sensing principle

The level of lactate was obtained by monitoring the activity of the LOx that, in presence of oxygen, catalyzes the oxidation of lactate to pyruvate with the simultaneous reduction of oxygen (O_2_) to hydrogen peroxide (H_2_O_2_). The H_2_O_2_ can be monitored electrochemically to obtain an amperometric output that is directly proportional to the lactate concentration. The peroxidase activity of PB increases the catalysis toward the reduction of H_2_O_2_ to H_2_O while simultaneously undergoing an electrochemical redox reaction to Prussian white (PW) (Fig. 1) [31]. Briefly, the working area of the electrode, modified with the hybrid nanocomposite, was covered with 100 µL of phosphate buffer at pH 7.2 containing different concentrations of lactate. After 3 min, the enzymatic activity was calculated by measuring the amount of the electroactive byproduct derived from the reduction of H_2_O_2_. A chronoamperometric technique at − 0.1 V (versus Ag/AgCl) for 60 s was used to monitor the enzymatic activity, and the current intensity of the chronoamperograms was recorded in the presence and absence of the substrate, highlighting an increase as the lactate concentration increases (Fig. 1).

### 3D-printed wearable device

The 3D-printed flexible wearable device was designed using the Autodesk Fusion 360 3D-design application and printed with the Creality Ender-3 V2 Neo 3D printer (Creality 3D Technology, Shenzhen, China). Transparent thermoplastic polyurethane (TPU) (Eryone Technology Co., Shenzhen, China) was used for fabricating the flexible wearable device. The device features a flexible and adjustable design, with belt-like straps allowing precise size customization to fit various arm sizes comfortably and securely. The dimensions of the 3D-printed armband are presented in Fig. S1 in the Supplementary Information (SI). To ensure the stability of the electrochemical sensor during intense physical activity, the device includes a designated slit for a USB cable connection, enabling real-time monitoring of lactate levels in sweat using a smartphone (Fig. 5). The transparent TPU material used for printing the wearable sensor ensured the continuous and reliable operation of the potentiostat, with a clear light indicator (blue for connected device, red during the measurement process) confirming its proper functioning (Fig. 5). Electrochemical measurements were performed using the Sensit Smart portable potentiostat (PalmSens, Houten, The Netherlands), which was connected to a smartphone running the open-source mobile application PStouch by PalmSens BV. Sweat sampling was achieved through capillary action, using the integrated SPE sensor combined with filter paper. The analysis of the sweat sample was conducted via the chronoamperometric method, as detailed in the previous section.

### LC–MS/MS lactate analysis

A stock solution of 10 mg of lactate was prepared by dissolving the analyte in Milli-Q water in a volumetric flask and diluting to the mark to obtain a standard solution of 1 mg/mL lactate. The stock solution was stored at − 20 °C until analysis. Quantitative analysis was conducted by constructing calibration curves. Standard mixtures were prepared by serial dilutions to achieve the following concentrations: 100, 200, 400, 600, and 800 µg/L. Samples were diluted 1000-fold by serial dilution using Milli-Q water. The diluted samples were directly transferred into the HPLC auto-sampler, and 1 µL of the supernatant was analyzed using an LC–MS/MS method. Lactate detection was performed on an Agilent 1200 high-performance liquid chromatograph coupled with an Agilent 6420 triple-quadrupole mass spectrometer (Agilent Technologies, USA). The mass spectrometer was operated in negative electrospray ionization (ESI) mode. The drying gas temperature was set to 300 °C, with a flow rate of 0.200 mL/min, a nebulizer pressure of 0.35 MPa, and a capillary voltage of 4000 V. Chromatographic separation was achieved using a Phenomenex Kinetex C18 column (2.1 × 100 mm, 5 µm). The mobile phases consisted of water (A) and acetonitrile (B). The gradient was programmed as follows: 5% B for 2 min, increased to 80% B over 1 min, held for 3 min, and returned to 5% B within 1 min, followed by a 2-min post-run equilibration. For Multiple Reaction Monitoring (MRM), lactate was monitored using the precursor ion at *m/z* 89 and following two transitions at *m/z* 45.1 and *m/z* 43.3. The dwell time for each transition was set to 200 ms, and the collision energy was 15 eV for both transitions. Negative ionization mode was used for all measurements.

## Results and discussion

### Optimization of the enzyme-based system for lactate monitoring

To demonstrate the successful modification of the material, we performed SEM and EDX to characterize the modified electrode surface and confirm the successful deposition of the sensing materials. The SEM images illustrate the morphological aspect after each modification step, while the EDX analysis verifies the elemental composition corresponding to the added materials. These results confirm that the presence of the CB layer and especially the PB makes the electrode more conductive and makes it easier to characterize with the instrument. In fact, the surface of the bare electrode is instantly burned, damaging the sample. On the other hand, a more homogeneous structure was observed with the presence of CB and PB. Furthermore, the presence of the PB layer was evaluated through EDX, where it is possible to notice (Fig. S2B) the presence of Fe at approximately 7%. The element, however, is not detected in the other two electrode samples, confirming the correct fabrication of the sensors. These details have been included in the revised SI (see Fig. S2A, B). As a starting point, the quality of the handmade printed strips, modified with CB/PB dispersion (SPE-CB/PB) and the peroxidase-like activity of PB toward the reduction of H_2_O_2_ to H_2_O, were evaluated. Cyclic voltammetry (CV) was used as the electrochemical technique for characterization in phosphate buffer, pH 7.2 and in presence of 50 mU of LOx and 1 mM lactate. When the substrate was added, the increase of the anodic and cathodic peak currents indicated the catalytic effect of PB on H_2_O_2_ reduction, produced by the previous oxidation of lactate catalyzed by LOx [31, 35], as can be seen in the cyclic voltammograms reported in the supplementary material, Fig. S3. Figure S4 presents chronoamperometric data for the enzyme/CB/PB electrodes, both with and without lactate, demonstrating the sensor’s lactate response and emphasizing PB’s contribution to lactate detection. Next, the optimization of the CB/PB dispersion volume to be drop-cast on the WE surface was evaluated up to 8 µL. The optimization was performed using the CA technique by applying a constant potential of − 0.1 V for 60 s. Measurements were performed in triplicate (*n* = 3) using 50 mU of LOx enzyme and 1 mM of lactate substrate in phosphate buffer, pH 7.2. The histograms in Fig. S5 show, for each CB/PB volume evaluated, the signal variation observed in the comparative analysis between the current intensities from the enzyme–substrate reaction and the blank (without lactate). The most significant signal change was observed when the electrodes were modified with 2 µL of CB/PB dispersion which allowed obtaining a good repeatability and the highest catalytic action toward the reduction of H_2_O_2_.

**Fig. 2:**
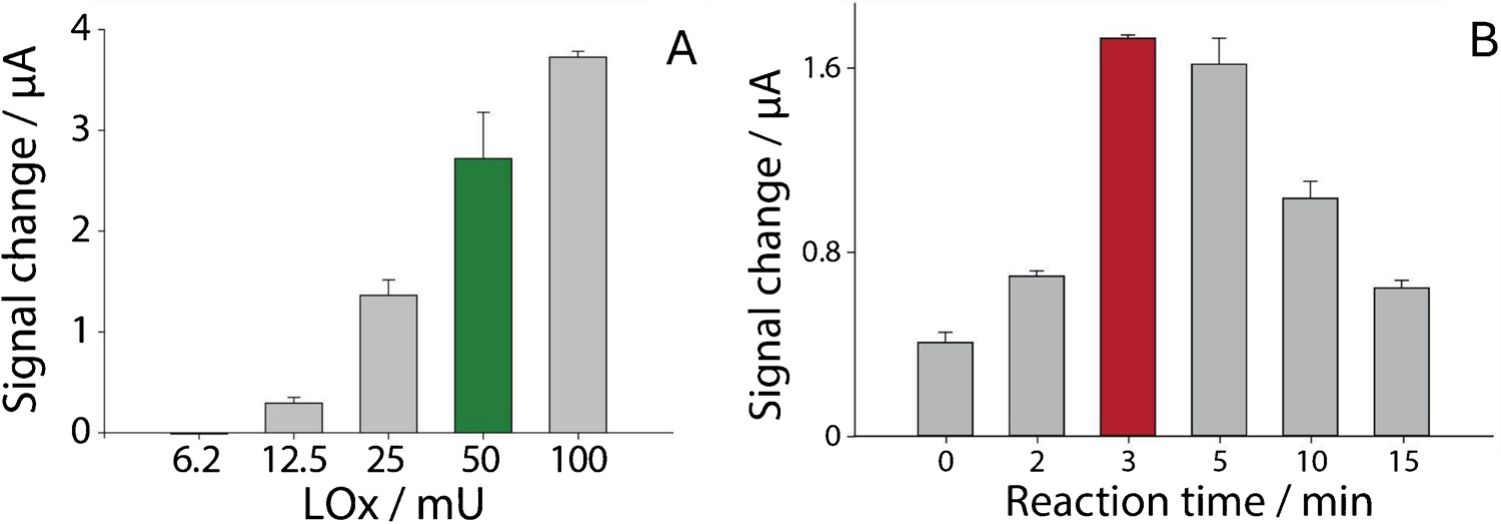
**A** Optimization of the amount of LOx capable of catalyzing the reaction, in the range from 6.5 to 100 mU, in the presence of 1 mM of lactate. **B** Optimization of the reaction time when 2 µL of the enzyme (50 mU) was immobilized onto the electrode surface, evaluated from 0 to 15 min, in the presence of 100 µL drop of 1 mM of lactate. Measurements were performed in triplicate (*n* = 3)

To reflect the disposable nature of the sensor, each measurement was performed using a freshly prepared electrode from a different modification batch, demonstrating the reproducibility and robustness of the fabrication process across multiple independent preparations. The developed biosensor used a lactate oxidase (LOx)-modified sensing electrode to detect lactate concentration by the chronoamperometric method. The optimal amount of LOx enzyme for catalyzing the reaction was determined by testing different enzyme dilutions in the range from 6.25 to 100 mU. Measurements were conducted in the presence of 1 mM lactate, on three replicates. As shown in Fig. 2A, the best amount of LOx was found to be 50 mU, chosen as a compromise between the amount of enzyme and the current generated by the reaction, since increasing the amount of enzyme did not result in a proportional increase in current. The lack of a proportional increase in current when increasing the enzyme amount can be explained by the limitations in electron transfer efficiency. As the amount of enzyme increases, it is possible that the electron transfer to the electrode surface becomes less efficient due to steric hindrance or the enzyme molecules being less accessible to the electrode surface. A higher enzyme load may also lead to a denser enzyme layer, which could hinder the optimal interaction between the enzyme and the target substrate, reducing the efficiency of the electrochemical reaction. This phenomenon, where increasing enzyme concentration does not lead to a linear increase in current, is consistent with observations in enzyme-based electrochemical sensors, where an optimal enzyme loading exists [36].

Accordingly, the electrode was modified with 2 µL of a 25 U/mL LOx solution (50 mU on the electrode) and different reaction times, in the range from 0 to 15 min, were tested (Fig. 2B). Measurements were carried out with a 1 mM lactate solution prepared in phosphate buffer, pH 7.2. The most significant signal change was observed waiting a reaction time of 3 min, which allowed obtaining a good repeatability and the highest catalytic action toward the reduction of H_2_O_2._ The signal decrease observed after 5 min may be due to enzyme desorption from the surface, substrate depletion, or by-product accumulation, as reported in similar systems. However, this does not affect sensor applicability, as the device is intended for short-term, single use monitoring rather than continuous long-term operation [37, 38].

### Analytical performance

**Fig. 3:**
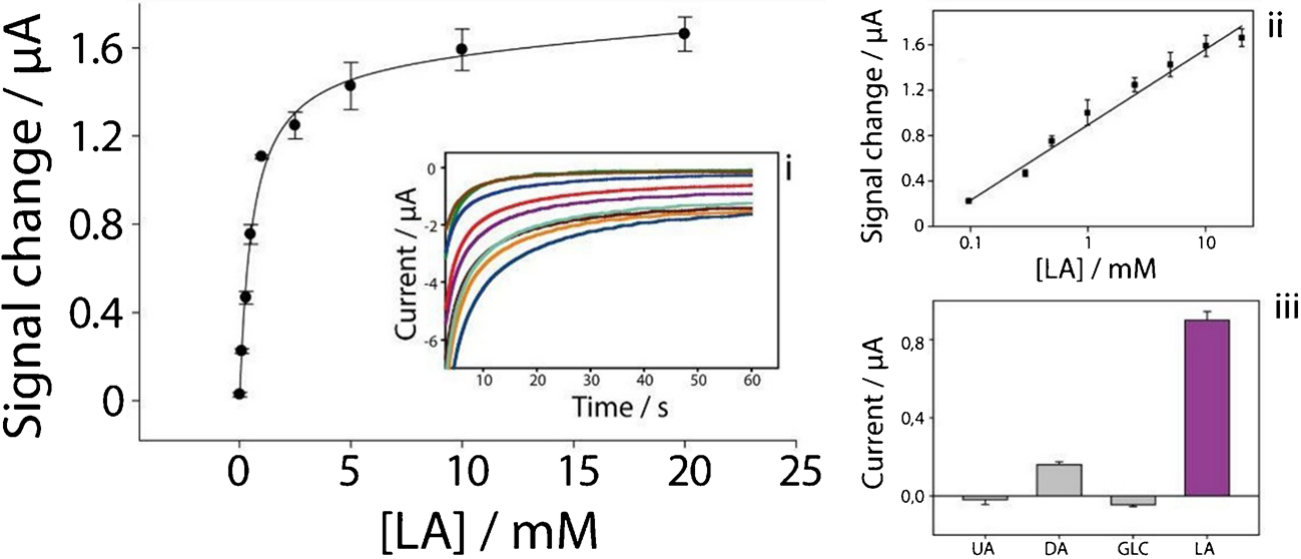
Calibration curve obtained at increasing concentrations of lactate (LA), evaluated from 0.01 to 20 mM, in the presence of 50 mU of Lox immobilized onto the electrode surface, with a reaction time of 3 min; inset i—chronoamperometric curves recorded at − 0.1 V for 60 s; inset ii—regression line in the range from 0.1 to 20 mM (logarithmic scale on the *x*-axis), with the corresponding linear equation. Measurements were performed in triplicate (*n* = 3); inset iii—interference study: chronoamperometric measurements in the presence of 1 mM concentration of lactate, uric acid (UA), dopamine (DA), and glucose (GLC)

The analytical performance of the system was studied at increasing lactate concentrations (0.01–20 mM), when the enzyme was immobilized on the electrode surface, with a reaction time of 3 min. An exponential trend can be observed, reaching a plateau starting from a lactate concentration of 2.5 mM (Fig. 3).

A linear range can be identified from 0.1 to 20 mM of lactate, when the signal change is reported as a function of the logarithm of the concentration and it is described by the equation *y* = 0.67*log*x* + 0.89 (*R*^2^ = 0.99) as shown in the inset ii of the Fig. 3. The repeatability, calculated as the relative standard deviation (RSD%), was found to be 5%, with a detection limit of ca. 60 µM calculated as 3·*σb*/*m* (where *σb* is the standard deviation of the blank signals and *m* the slope of the regression line) while the limit of quantification, calculated as 10·*σb*/*m*, was found to be approximately 200 µM. To confirm the specificity of the sensor, we tested it in the presence of uric acid (UA), dopamine (DA) and glucose (GLC) [39], all at a concentration of 1 mM in the phosphate buffer pH 7.2. The inset iii of the Fig. 3 showed no significant signal from any interferents compared to 1 mM lactate, noting that dopamine exhibits low current intensity despite being tested at high concentrations relative to physiological values in sweat [40]. This indicates that the sensor is highly selective toward lactate molecules.

### Application on real sweat samples using the 3D-printed device

**Fig. 4:**
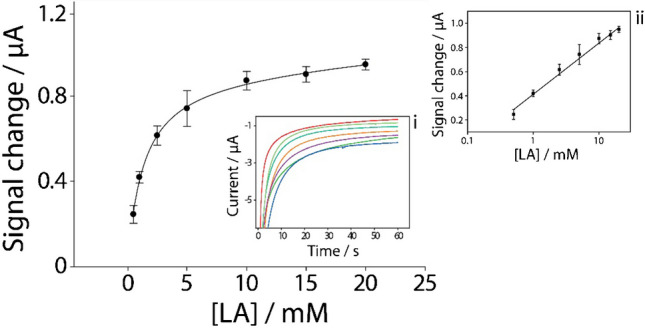
Calibration curve in undiluted sweat at increasing concentrations of lactate, evaluated from 0.5 to 20 mM, in presence of 50 mU of Lox immobilized onto the electrode surface, with a reaction time of 3 min; inset i—chronoamperometric curves recorded at − 0.1 V for 60 s; inset ii—regression line in the range from 0.5 to 20 mM (logarithmic scale on the *x*-axis) with the relative linear equation. Measurements were performed in triplicate (*n* = 3)

The actual contribution of the microfluidic channel to sweat collection and transportation and the influence on the accuracy and reliability of the detection results was then evaluated. In Fig. S6, a comparison of the sensor chronoamperometric response to 5 mM lactate in sweat with and without the paper-based microfluidic channel is presented, demonstrating the improved consistency and stability of the detection signal when the microfluidic channel is used, which can be attributed to a more controlled sweat transport and uniform sample delivery [28, 29]. The analytical performance of the combined system was studied in undiluted sweat, by adding increasing concentrations of lactate, from 0.5 to 20 mM, with the enzyme immobilized on the electrode surface and a reaction time of 3 min. It is possible to observe an exponential trend, reaching a plateau starting from a lactate concentration of 5 mM (Fig. 4). A linear range can be identified in the 0.5–20 mM range when the signal change is reported as a function of the logarithm of the concentration and it is described by the equation *y* = 0.42*log*x* + 0.41 (*R*^2^ = 0.98), as shown in the inset ii of the Fig. 4. The repeatability of the entire system, calculated as RSD%, was found to be 6%, with the detection limit of 0.2 mM and a LOQ of 0.8 mM.

Following the limit of detection (LOD) calculation, we compared our method to existing methods in terms of linear range, LOD, and matrix used, as shown in Table S1. This comparison highlights the performance of our amperometric method, which demonstrates a competitive LOD of 0.2 mM in undiluted human sweat in real-time. Compared to other methods, such as reverse-phase high-performance liquid chromatography (RP-HPLC), Electrochemical Impedance Spectroscopy (EIS), and colorimetric analysis, our method offers comparable or superior sensitivity, particularly in monitoring lactate levels directly from sweat [41–44]. Additionally, Table S2 provides a summary of various enzyme-based wearable sensors applied to monitor lactate levels from different body parts. Notably, our 3D-printed TPU band-based sensor, which utilizes carbon black and Prussian blue, shows a similar linear range and LOD to other wearable devices, such as those used on the foot and thigh, while offering the added advantage of a customizable, flexible design suitable for continuous real-time monitoring. These comparisons emphasize the versatility and efficiency of our approach in wearable sweat lactate sensing applications.

**Fig. 5:**
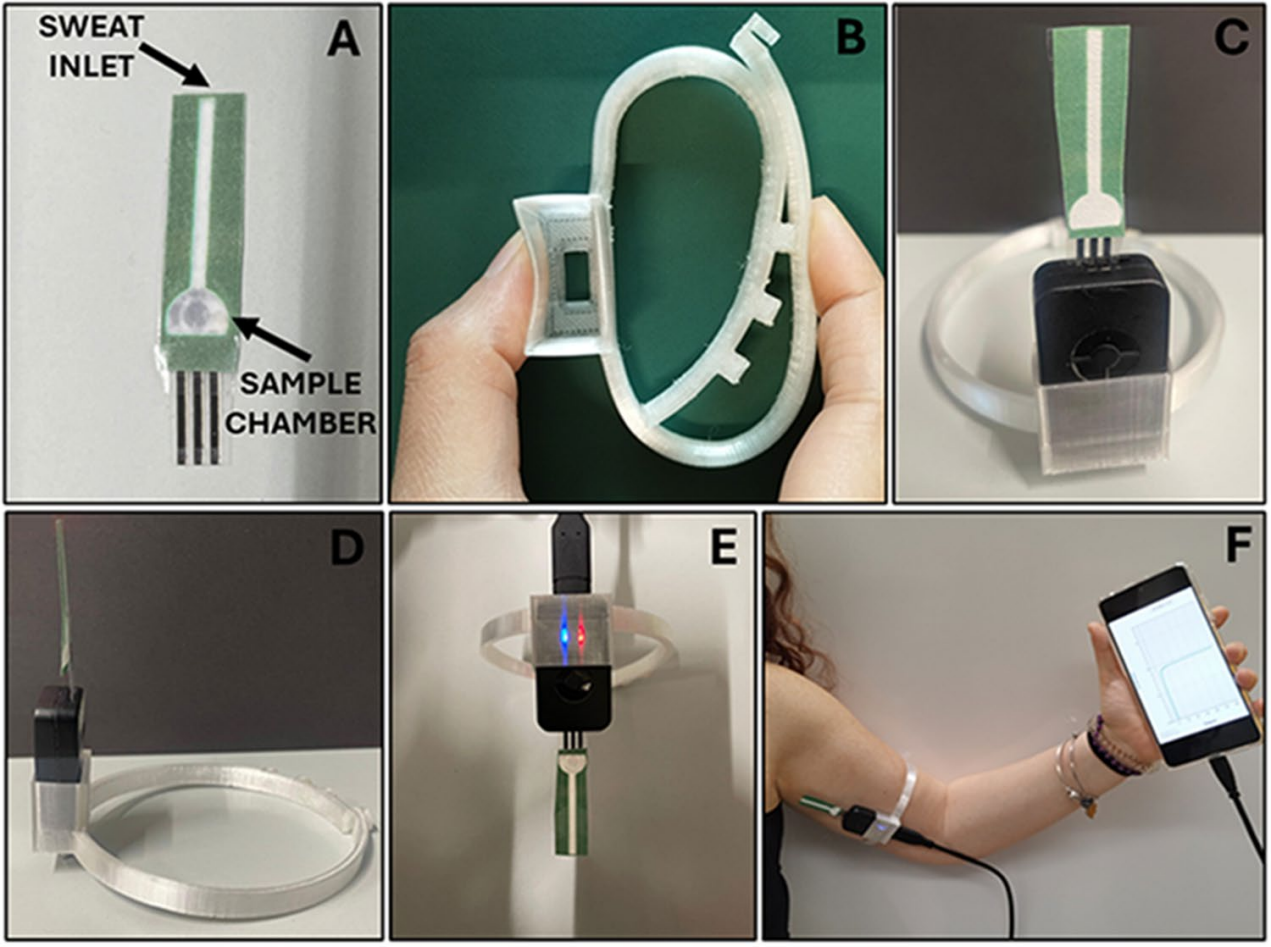
**A** SPE combined with the paper channel strip. **B** Wearable bracelet flexibility made of 3D-printed thermoplastic polyurethane (TPU). **C**,** D** The portable potentiostat was inserted into the 3D-printed wearable device through the designated slit. **E** Transparent TPU filament was selected to enable continuous monitoring of the potentiostat’s proper functioning through its light indicators. **F** 3D-printed wearable device integrated with the potentiostat and biosensor for real-time lactate detection in sweat

Consequently, the biosensor has been integrated, including the paper-based channel, into the customized 3D-printed architecture, as shown in Fig. 5.

The flexible 3D-printed wearable device, i.e., armband, was provided of a belt-like straps allowing customization of the wearable device according to the individuals’ arms sizes. The use of 3D-printed TPU ensures a lightweight, flexible and ergonomically contoured armband (Fig. 5B). The slit for the portable potentiostat enabled its insertion providing stability during the measurement process (Fig. 5C–F).

In order to validate the developed system, six sweat samples were collected from volunteers (A, B, C, D, E, F) and quantified. The results obtained were compared against the standard LC–MS/MS laboratory technique, obtaining a good correlation, between 94 and 103%, as shown in Table 1.

**Table 1:** Correlation between the lactate concentrations detected in the six different samples (A, B, C, D, E, F) analyzed by SPEs integrated with the paper channel strips, and standard LC–MS/MS standard method

Sample	SPE	LC–MS/MS	Correlation %
A	14.0 mM	13.9 mM	101%
B	14.6 mM	14.4 mM	101%
C	19.4 mM	20.2 mM	96%
D	13.8 mM	13.9 mM	99%
E	18.1 mM	17.5 mM	103%
F	11.8 mM	12.5 mM	94%

**Fig. 6:**
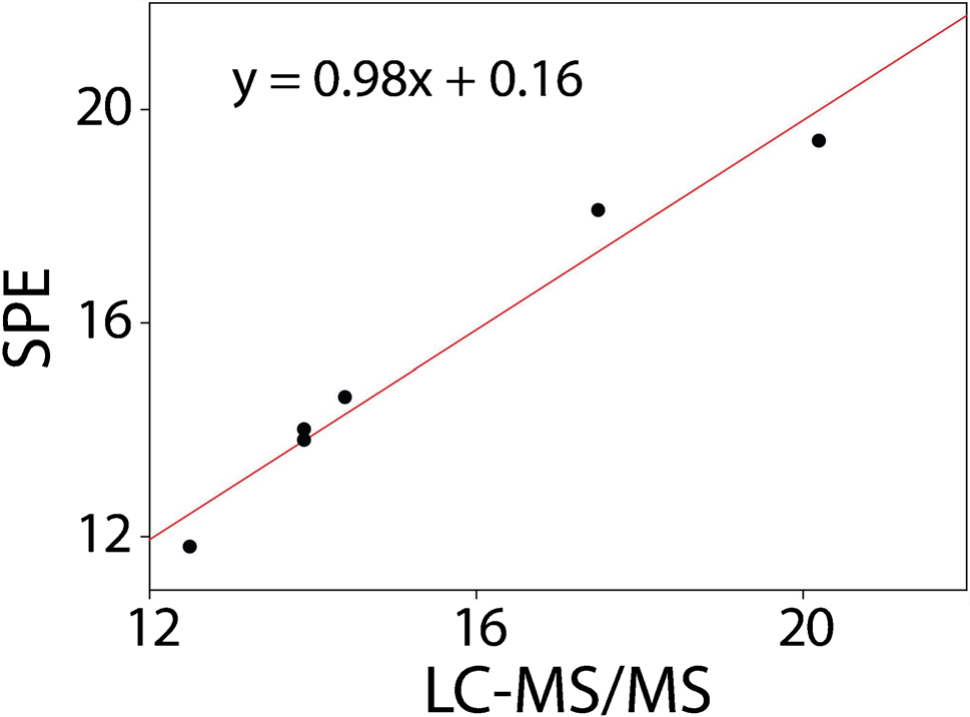
Correlation between lactate concentrations measured using the electrochemical biosensor and LC–MS/MS method for six real sweat samples. The red line represents the linear regression fit (*y* = 0.98*x* + 0.16, *R*^2^ = 0.96)

To assess the agreement between the electrochemical biosensor and the standard LC–MS/MS method, statistical analyses were performed: a Pearson correlation analysis revealed a strong positive linear relationship between the concentrations measured by the SPEs and those obtained via LC–MS/MS, with a correlation coefficient (*r*) of 0.98. Linear regression further supported this relationship, yielding a coefficient of determination (*R*^2^) of 0.96 (Fig. 6). This indicates that approximately 96.5% of the variance observed in the LC–MS/MS measurements can be explained by the values obtained using the SPEs. The correlation was found to be statistically significant (*p* = 4.702 × 10⁻^4^), confirming a high degree of agreement between the two analytical methods at the 0.05 significance level.

## Conclusions

In this work, a flexible electrochemical lactate biosensor on screen-printed polyester electrodes, modified with carbon black, Prussian blue, and lactate oxidase for chronoamperometric analysis of sweat was developed. Subsequently, to enable detection using the 3D wearable device, the screen-printed electrodes were integrated with paper channels for collecting the sweat sample from the skin and directing it toward the electrode’s detection surface. The sensor achieved a detection limit of 0.06 mM under optimized conditions and 0.2 mM in real sweat samples, with good correlation (94–103%) to the LC–MS/MS gold standard. Integration with a 3D-printed TPU bracelet enabled efficient sweat collection and transport, combining lightweight durability with a customizable, ergonomic design suitable for dynamic activities. This system demonstrates a cost-effective, sustainable, and non-invasive approach to continuous and personalized lactate monitoring, advancing wearable health technologies.

## Supplementary Information

Below is the link to the electronic supplementary material.

Supplementary file1 (DOCX 631 KB)

## Data Availability

The data generated and analyzed during the current study are available from the corresponding author upon request.
