# Supplementary material for: On-body electrochemical measurement of sweat lactate with the use of paper-based fluidics and 3D-printed flexible wearable biosensor
Source: Anal Bioanal Chem. 2025 May 9;417(17):3825–34. doi: 10.1007/s00216-025-05905-0 (PMC12227461; doi:10.1007/s00216-025-05905-0)
Supplement: Supplementary file 1 — Supplementary file1 (DOCX 631 KB) [file 216_2025_5905_MOESM1_ESM.docx]

**Supplementary Information**

**On-body electrochemical measurement of sweat lactate with the use of paper-based fluidics and 3D-printed flexible wearable biosensor**

Gabriella Iula^1+^, Antonella Miglione^1+*^, Panagiota M. Kalligosfyri^1^, Michele Spinelli^2^, Angela Amoresano^2^, Concetta Di Natale^3^, Ibrahim A. Darwish^4^, Stefano Cinti^1,5,6*^

^1^Department of Pharmacy, University of Naples ‘Federico II’, Via D. Montesano 49, 80131, Naples, Italy

^2^Department of Chemical Sciences, University of Naples “Federico II”, 80126 Naples, Italy

^3^Department of Chemical Materials and Industrial Production (DICMaPI), University of Naples Federico II, P.le Tecchio 80, Naples 80125, Italy

^4^Department of Pharmaceutical Chemistry, College of Pharmacy, King Saud University, P.O. Box 2457 Riyadh 11451, Saudi Arabia

^5^Bioelectronics Task Force at University of Naples Federico II, Via Cinthia 21, Naples 80126, Italy.

^6^Sbarro Institute for Cancer Research and Molecular Medicine, Center for Biotechnology, College of Science and Technology, Temple University, Philadelphia, USA.

+ These authors contributed equally.

*Corresponding authors: [antonella.miglione@unina.it](mailto:antonella.miglione@unina.it); [stefano.cinti@unina.it](mailto:stefano.cinti@unina.it)

Table S1: Comparison between the proposed chronoamperometric method and previously reported methods.

| **Detection Method** | **Linear range / mM** | **LOD / mM** | **Matrix** | **Ref** |
| --- | --- | --- | --- | --- |
| RP-HPLC | 0.3 - 350 | 0.03 | Human sweat stored at -20 °C until analysis | [41] |
| Colorimetric analysis / alginate beads | 10 -100 | 6.4 | Artificial sweat | [[42]](https://doi.org/10.3390/bios11100379) |
| EIS | 0.1–100 | 0.1 | Human sweat stored at -20 °C until analysis | [43] |
| Impedimetric | 3 - 100 | 1.5 | Human sweat twice diluted with phosphate buffer | [44] |
| Amperometry | 0.5-20 | 0.2 | Humans undiluted sweat in real time | This work |

RP-HLPC: Reversed-Phase High-Performance Liquid Chromatography, EIS: Electrochemical Impedance Spectroscopy

**Table S2:** Representative examples of enzyme-based wearable sensors applied for sweat lactate monitoring on various body parts.

| **Device /body part** | **WE modification** | **Detection Method** | **Linear range /**  **mM** | **LOD / mM** | **Ref** |
| --- | --- | --- | --- | --- | --- |
| Tattoo/arm | N/A | Amperometry | 1-30 | N/A | [17] |
| Eyeglasses/nose | Carbon black and Prussian blue | Amperometry | 1-14 | 0.39 | [20] |
| Sock/BFCs-foot | Chitosan | Linear polarization | 5-20 | 0.3 | [21] |
| Patch/thigh | Prussian Blue | Amperometry | 1-20 | 0.12 | [22] |
| 3D-printed TPU Band / arm | Carbon black and Prussian blue | Amperometry | 0.5-20 | 0.2 | This work |

N/A: Not applied, BFCs: biofuel cells, TPU: thermoplastic polyurethane


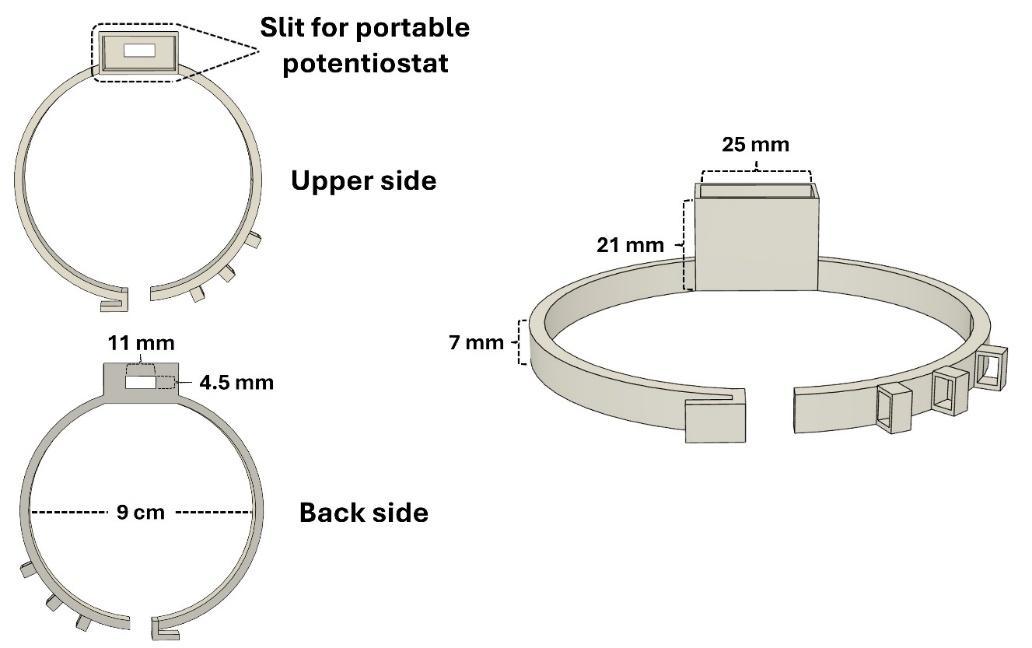


**Fig.S1** Dimensions of the flexible 3D-printed wearable device, i.e. armband. The belt-like straps allowed customization of the wearable device according to the individuals’ arms sizes. The slit for the portable potentiostat enabled its insertion providing stability during the measurement process.


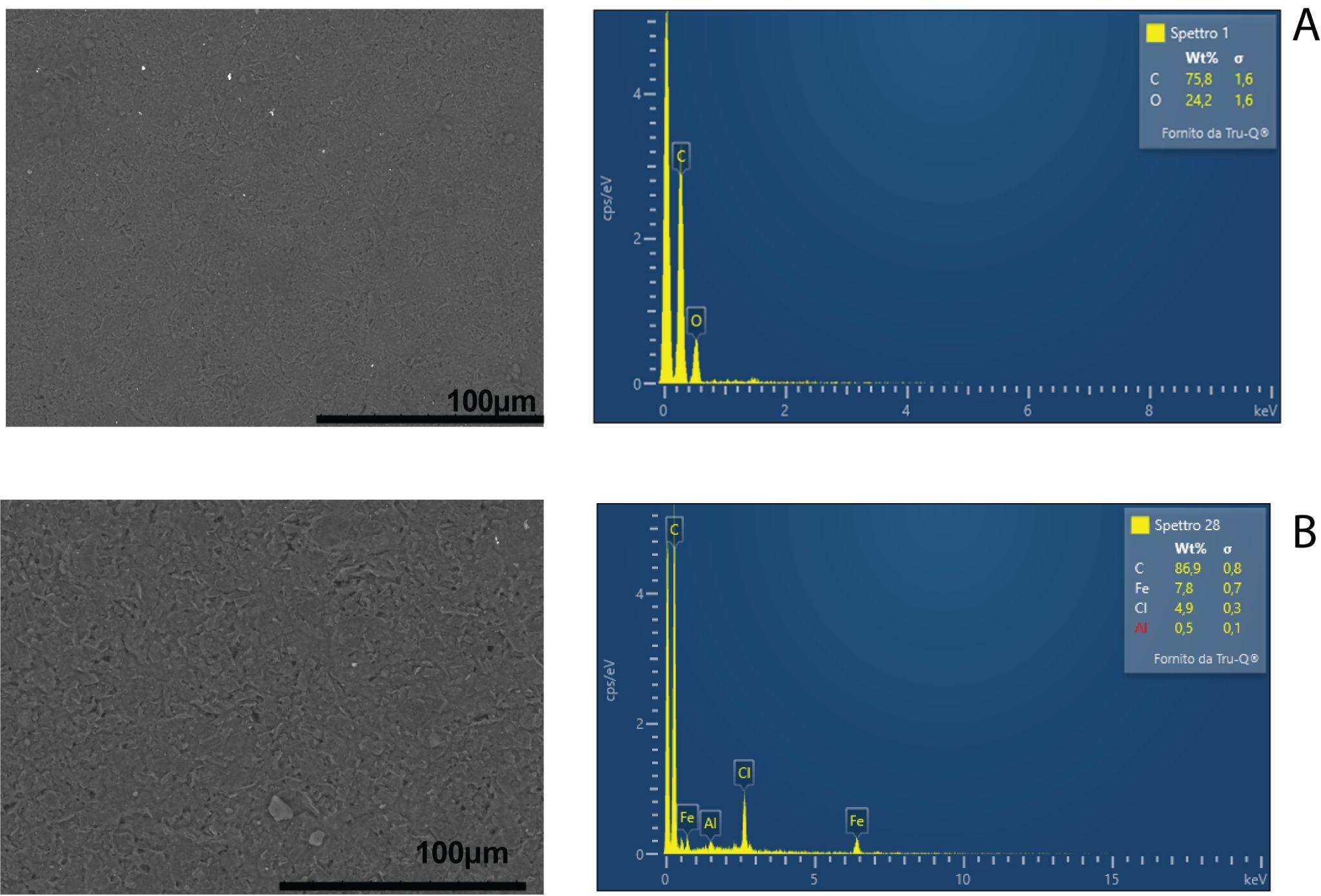


**Fig.S2** SEM and EDX characterization of the electrode surface after each modification step. A) Bare electrode; EDX analysis shows no detectable Fe signal. B) Electrode modified with the CB/PB dispersion, displaying a more uniform morphology and enhanced structural integrity. EDX confirms successful deposition of PB through the presence of Fe (~7%), indicating proper fabrication and improved conductivity of the sensing surface.


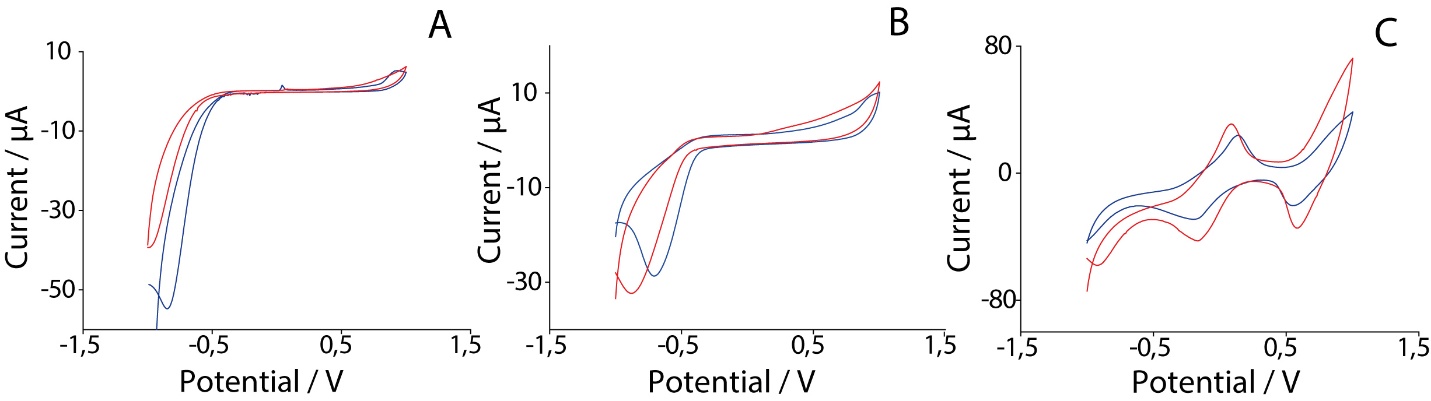

**Fig.S3** Cyclic voltammograms of A) bare electrode; B) CB electrode; C) CB/PB electrode in presence (red line) and absence (blue line) of H_2_O_2._


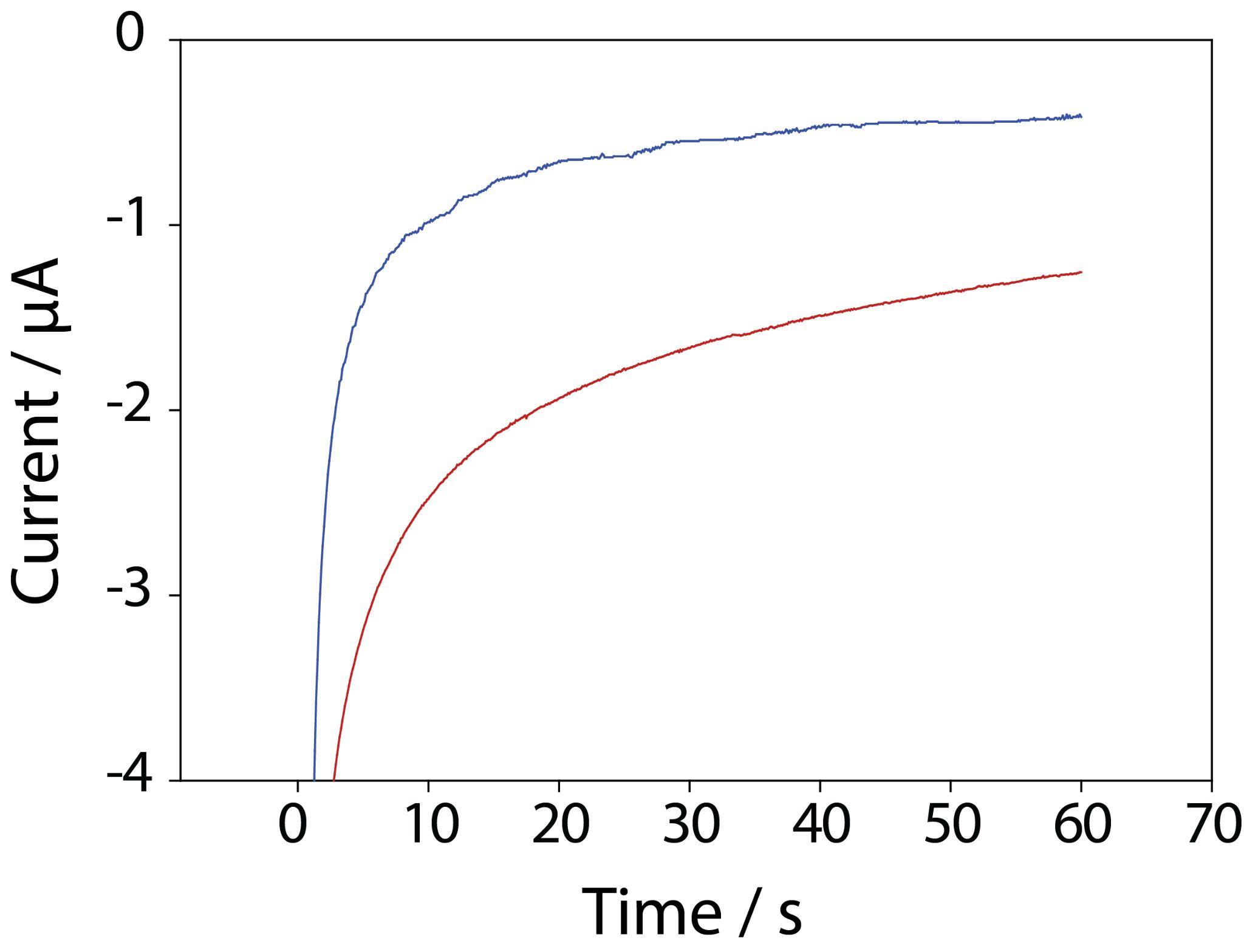


**Fig.S4** Chronoamperometric responses of enzyme/CB/PB electrodes in absence (blue line) and in presence (red line) and of lactate. The observed current increase in the presence of lactate highlights the sensor’s responsiveness and confirms the crucial role of Prussian blue in facilitating electron transfer for effective lactate detection.


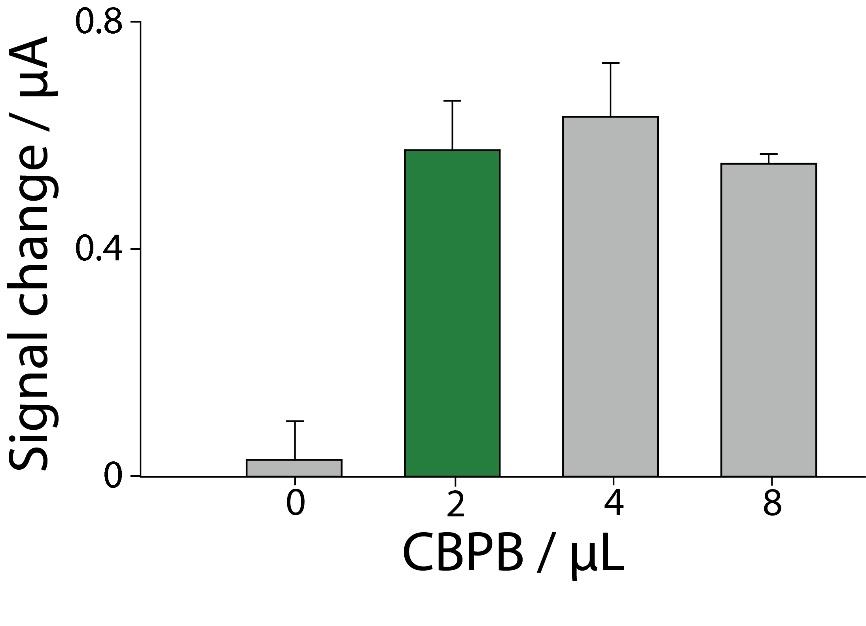


**Fig.S5** Optimization of the CB/PB dispersion volume to be drop-cast on the WE surface, evaluated from 0 to 8 µL, in presence of 50 mU of LOx and 1 mM of lactate. Measurements were performed in triplicate (n=3).


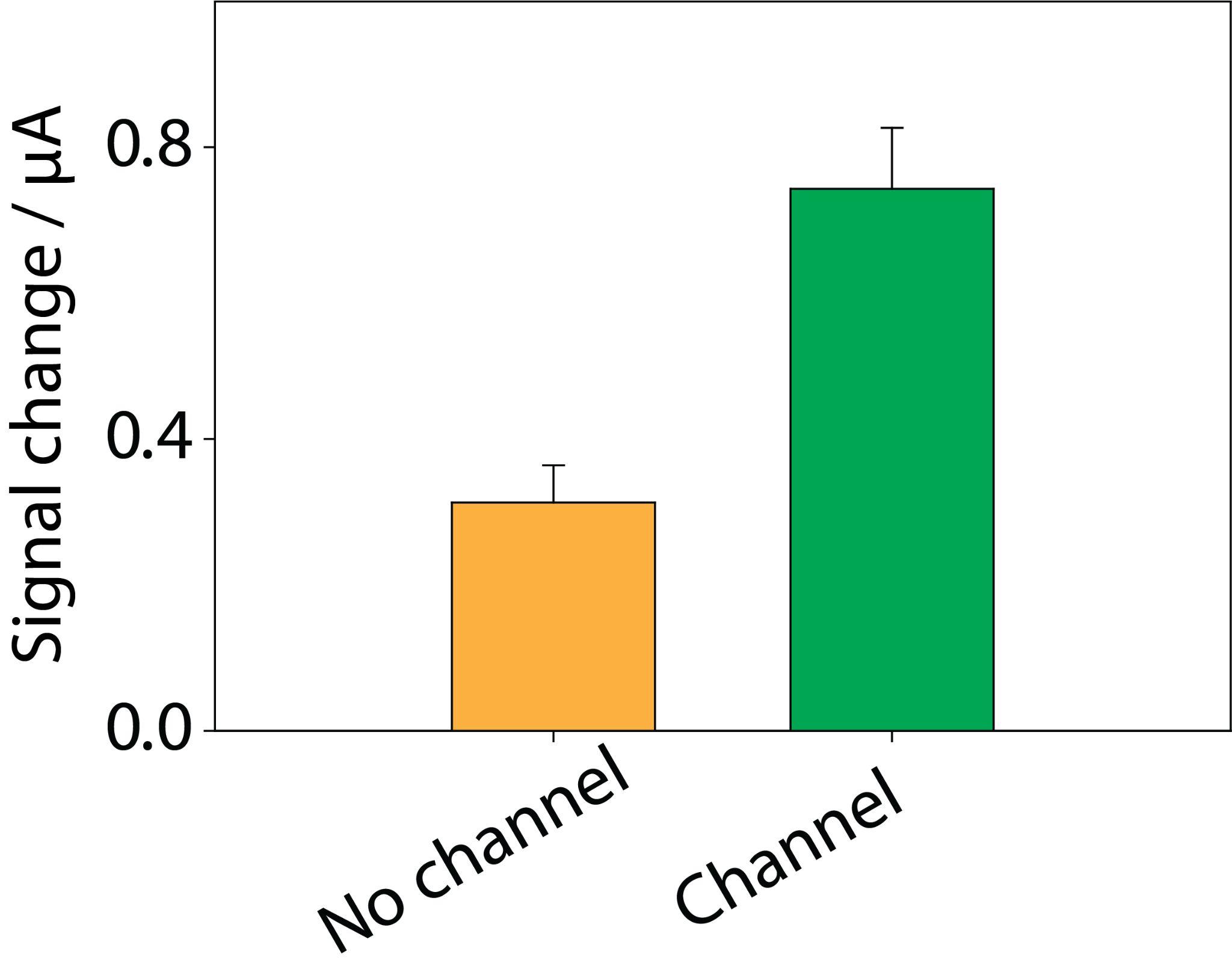


**Fig.S6** Comparison of the sensor chronoamperometric response to 5 mM lactate in sweat with and without the paper-based microfluidic channel. The data are presented as the signal difference between sweat alone and after the addition of 5 mM lactate.
